# Characterization and subcellular localization of Alongshan virus proteins

**DOI:** 10.3389/fmicb.2022.1000322

**Published:** 2022-09-27

**Authors:** Yinghua Zhao, Ping Wu, Li Liu, Baohua Ma, Mingming Pan, Yuan Huang, Nianyan Du, Hongyan Yu, Liyan Sui, Ze-Dong Wang, Zhijun Hou, Quan Liu

**Affiliations:** ^1^College of Wildlife and Protected Area, Northeast Forestry University, Harbin, China; ^2^Department of Infectious Diseases, Center of Infectious Diseases and Pathogen Biology, Key Laboratory of Organ Regeneration and Transplantation of the Ministry of Education, The First Hospital of Jilin University, Changchun, China; ^3^School of Life Sciences and Engineering, Foshan University, Foshan, China

**Keywords:** segmented flavivirus, Alongshan virus, viral proteins, characterization, subcellular localization

## Abstract

Alongshan virus (ALSV) in the Jingmenvirus group within the family *Flaviviridae* is a newly discovered tick-borne virus associated with human disease, whose genome includes four segments and encodes four structural proteins (VP1a, VP1b, VP2, VP3, and VP4) and two non-structural proteins (NSP1 and NSP2). Here, we characterized the subcellular distribution and potential function of ALSV proteins in host cells. We found that viral proteins exhibited diverse subcellular distribution in multiple tissue-deriving cells and induced various morphological changes in the endoplasmic reticulum (ER), and NSP2, VP1b, VP2, and VP4 were all co-localized in the ER. The nuclear transfer and co-localization of VP4 and calnexin (a marker protein of ER), which were independent of their interaction, were unique to HepG2 cells. Expression of NSP1 could significantly reduce mitochondria quantity by inducing mitophagy. These findings would contribute to better understanding of the pathogenesis of emerging segmented flaviviruses.

## Introduction

In recent years, various pathogenic tick-borne viruses have been identified to be associated with human febrile illness (Dong et al., 2021; Ma et al., 2021; Wang et al., 2021). Alongshan virus (ALSV), a segmented flavivirus in the Jingmenvirus group of the family *Flaviviridae*, has been detected in tick-bitted patients and *Ixodes persulcatus* in Northeast China (Wang et al., 2019). Subsequently, ALSV is also found in *Ixodes persulcatus* in Russia and in *Ixodes ricinus* in Finland (Kuivanen et al., 2019; Kholodilov et al., 2020), whose emergence may be associated with its codon usage (Rahman et al., 2022). The genome of ALSV is divided into four segments (S1–S4): S1 encodes flavivirus NS5-like non-structural protein 1 (NSP1), S2 encodes structural glycoproteins VP1a, VP1b and VP4, S3 encodes flavivirus NS2b3-like non-structural protein NSP2, and S4 encodes nucleoprotein VP2 and membrane protein VP3 (Wang et al., 2019; Zhang et al., 2020). The structural features of ALSV NSP2 is conserved in comparison with flavivirus NS3 helicases (Gao et al., 2020). Patients with ALSV infection mainly present with fever and headache, and some patients are accompanied by clinical symptoms of fatigue, coma, depression, nausea, myalgia/arthralgia, and skin rash (Wang et al., 2019). There is no effective vaccine and drug available for ALSV. A better understanding of the pathogenic mechanism of ALSV would contribute to the development of vaccines and antiviral drugs.

Flavivirus particles enter cells *via* endocytosis, exposing them to an acidic endosome that triggers the release of viral RNA into the cytosol (Modis et al., 2004; Zhang et al., 2015). The released positive-sense RNA is recognized by ribosomes and translated at the rough endoplasmic reticulum (ER) into a single polyprotein, which is cleaved into structural and non-structural proteins by the viral and cellular proteases. The non-structural proteins orchestrate ER membrane rearrangement to form the single-membrane invaginations that house the replication machinery (Neufeldt et al., 2018). Then, the viral gRNA molecules generated by the viral replicase complex are incorporated into viral particles which involve RNA encapsidation and budding into the lumen of the ER (Welsch et al., 2009). Finally, the newly synthesized virus particles are transported from ER and Golgi apparatus to the cell surface, and released into extracellular by exocytosis (Xin et al., 2017). During the replication process, viruses manipulate the cellular organelles of the host to propagate and cause disease. Various flaviviruses, such as Zika virus (ZIKV), Dengue virus (DNEV), and hepatitis C virus (HCV), have evolved multiple strategies to drive cellular reprogramming by inducing a series of organelles (such as endoplasmic reticulum, Golgi apparatus, and mitochondria) structural and functional changes that promote viral replication (Neufeldt et al., 2018).

The subcellular trafficking with host cells and biological functions of ALSV proteins remain largely unknown. In the present study, we cloned all the genes of ALSV proteins into eukaryotic vectors for expression in various mammalian cells and examined their subcellular location using immunofluorescent assay. We found that in multiple tissue-deriving cells, viral proteins exhibited diverse subcellular distribution and induced various ER morphological changes, and NSP2, VP1b, VP2, and VP4 proteins were all co-localized with ER. The nuclear transfer and co-localization of VP4 and calnexin, which independent of their interaction was unique to HepG2 cells, and NSP1 reduced mitochondria quantity by inducing mitophagy. These findings would contribute to the understanding of the pathogenesis of emerging segmented flaviviruses.

## Materials and methods

### Cells and antibodies

Human embryonic kidney (HEK293T), human liver cancer (HepG2), and African green monkey kidney (Vero) cells were grown and maintained in Dulbecco’s Modified Eagle Medium (DMEM, high glucose, HyClone) containing 10% heat-inactivated FBS (BBI), 100 U/ml of ampicillin, and 100 g/ml of streptomycin (Sangon). Anti-Flag, Cox IV, calnexin, Histone, GM130, TIM23, TOM20, P62, B23, GAPDH, and CoraLite 594 or 488-conjugated IgG secondary antibodies were obtained from Proteintech (Rosemont, IL, United States). HRP-labeled goat anti-mouse or rabbit IgG were purchased from Nachuan Bio, and anti-LC-3b antibody was from CST.

### Plasmids construction and transfection

Flag-tagged ALSV NSP1, NSP2, VP1a, VP1b, VP2, VP3, and VP4 encoding genes were cloned into VR1012-based expression vector and confirmed by sequencing. Transfection of the plasmids into the indicated cells using Lipofectamine 2000 (Invitrogen) when the cells had grown to approximately 80% confluence.

### Immunofluorescence

Immunofluorescence was performed as previously described (Sui et al., 2021). HEK293T or HepG2 cells cultured on 12-mm coverslips were transfected with the indicated plasmids. After 24 h, cells were fixed with 4% paraformaldehyde and permeated with 0.5% Triton X-100. After cells were washed with PBST, they were blocked in 1% BSA and stained with primary antibodies, followed by staining with CoraLite 594 or 488-conjugated IgG secondary antibodies. Nuclei were stained with DAPI (Yesen Biotechnology, Shanghai, China). Fluorescence images were obtained and analyzed using a confocal microscope (FV3000, OLYMPUS).

### Co-immunoprecipitation and immunoblot analysis

Co-immunoprecipitation was performed as previously described (Liu et al., 2022). In brief, cells were lysed in the lysis buffer containing protease and phosphatase inhibitor cocktail (Selleck, Houston, Texas, United States). For co-immunoprecipitation, lysates were incubated overnight with ANTI-FLAG^®^ M2 Affinity Gel (Sigma-Aldrich). Then, proteins were separated by SDS-PAGE and electro-transferred onto the PVDF membrane. The membrane-containing proteins were blocked for 1 h at room temperature with 5% BSA in PBST, followed by treatment with the indicated primary antibodies overnight at 4°C. Subsequently, blots were incubated with secondary antibody for 1 h at room temperature and visualized *via* enhanced chemiluminescence reagents (Millipore, Billerica, MA, United States) with ChemiDoc XRS^+^ Molecular Imager software (Bio-Rad, Philadelphia, PA, United States).

### Nuclear and cytoplasmic extraction

Nuclear and cytoplasmic extraction was performed as previously described (Zhao et al., 2021). Briefly, cells were treated using the nuclear and cytoplasmic protein extraction kit (Beyotime, China) to prepare the nuclear and cytoplasmic fraction, according to the manufacturer’s instructions. The purified cytoplasmic and nuclear fractions were subjected to immunoblots assay according to the standard procedures with the relevant antibodies.

### Mitochondrial isolation

Total cells were lysed in the lysis buffer containing the protease and phosphatase inhibitor cocktail (Selleck, Houston, Texas, United States). Mitochondrial and cytosolic proteins were isolated using the Cell Mitochondrial Isolation Kit (Beyotime, China) and subjected to immunoblots according to the standard procedure with the relevant antibodies.

### Virus infection

ALSV, isolated from a tick-bitted patient in Northeast China (Wang et al., 2019), was used to infect the target cells. Briefly, the cultured cells were infected with ALSV at a multiplicity of infection (MOI) of 10 diluted in serum-free DMEM, and incubated for 2 h at 37°C. After washing three times with PBS, the infected cells were cultured with DMEM containing 2% FBS for the indicated times.

## Results

### Construction of expression vectors for ALSV proteins

**Figure 1:**
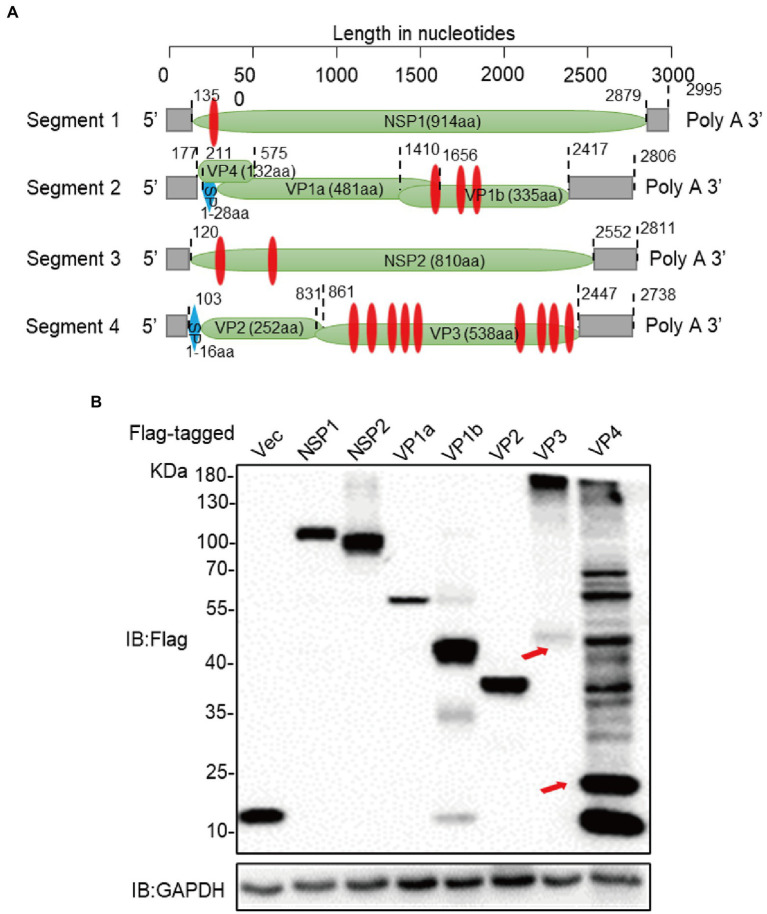
Construction of expression vectors for ALSV proteins. **(A)** Schematic genome organization and putative proteomic maps of ALSV, the open reading frame (ORF) is shown in the green box. The transmembrane area is displayed in red box, and the signal peptide is displayed in blue box. **(B)** Immunoblot analysis of ALSV protein expression. The ALSV proteins plasmids were separately transfected into HEK293T cells, and the Flag-vec was used as a control. At 24 h post-transfection (hpt), cellular lysis was measured by immunoblot with Flag antibody, GAPDH as a loading control. The red arrows, respectively, indicate VP3 and VP4 bands which consistent with the calculated molecular weights.

In order to clarify the function of these ALSV-encoded proteins (Figure 1A), we constructed Flag-tagged NSP1, NSP2, VP1a, VP1b, VP2, VP3, and VP4 proteins expression plasmids and transfected them into HEK293T cells. At 24 h post transfection (hpt), the whole cell lysis was suffered to immunoblot assay with anti-Flag antibody to confirm the expression of viral proteins. The observed bands of viral protein were close to the calculated molecular weights, but VP3 showed an obvious band above 130 KD and VP4 appeared multiple diffusely distributed bands (Figure 1B), indicating that they may undergo multimerization and/or post-translational modification.

### The ER localization of ALSV proteins in HEK293T cells

**Figure 2:**
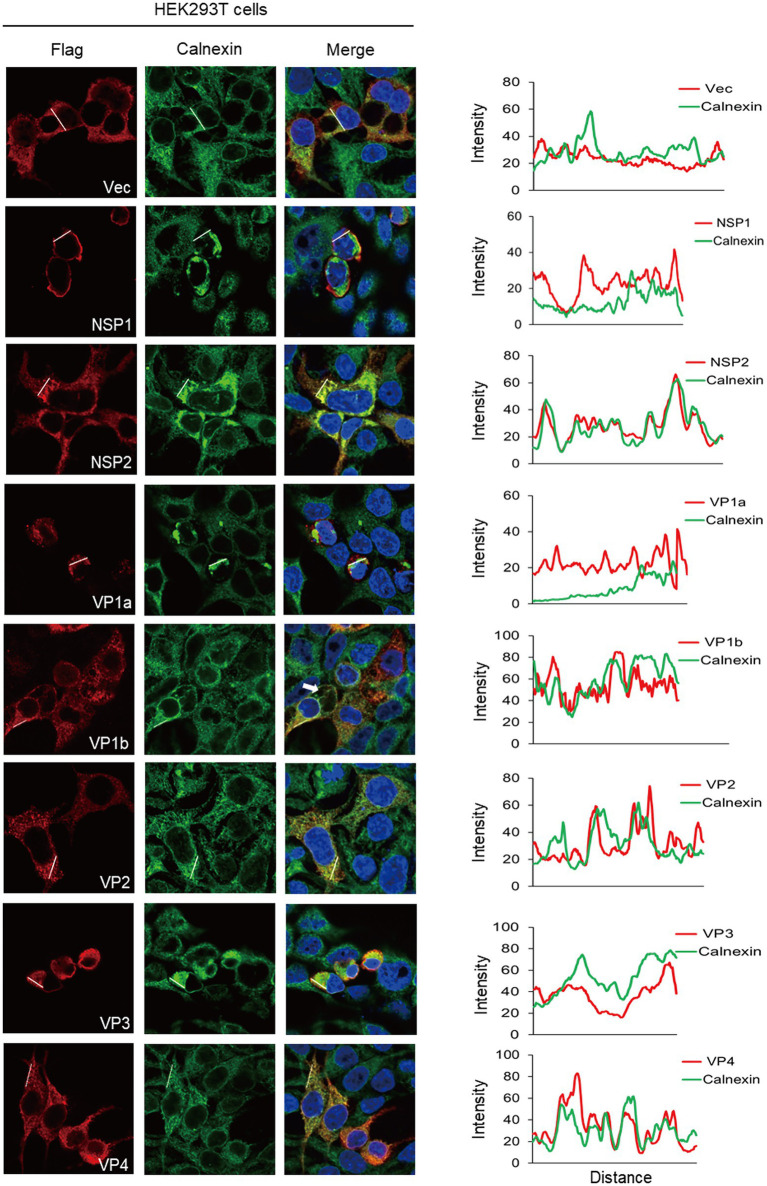
The ER subcellular localization of ALSV proteins in HEK293T cells. HEK293T cells were transfected with the plasmids expressing Flag-NSP1, NSP2, VP1a, VP1b, VP2, VP3, VP4 or vector. After 24 h, the cells were immune-stained with anti-Flag (red) and anti-calnexin (green) antibodies and counterstained with DAPI to examine chromosomes (blue). (Right) ImageJ line scan analysis of the intensity profiles of the viral protein (red) and the calnexin (green) along the plotted lines. The distance in the x-axis represents the length of the plotted lines, and the consistency of the change trend of intensity profiles is proportional to the degree of co-localization of the two proteins.

ER is the major cellular organelle that viruses need to usurp because it is a factory for producing viral proteins. First, we examined the locations of ALSV proteins with ER. Calnexin is an ER-residing chaperone protein and serves as a marker protein of ER, which is essential for controlling the production of glycosylated proteins in the process of viral infection and immune escape (Kohli et al., 2021). To this end, HEK293T cells were fixed at 24 hpt and stained with anti-Flag in red fluorescence and anti-calnexin to visualize the ER in green fluorescence. Consequently, we found that all ALSV viral proteins were distributed in the cytoplasm and associated with ER (Figure 2). The colors merge showed that the red fluorescence of NSP2 and VP4 was overlapping with the green fluorescence of calnexin, and the ImageJ line scan analysis found that the change trend of fluorescence intensity profiles of NSP2 and VP4 proteins was consistent with that of calnexin, which demonstrated that NSP2 and VP4 proteins significantly colocalized with ER. The changing trend of fluorescence intensity profiles of VP1b and VP2 proteins is consistent with Calnexin to some extent, indicating that VP1b and VP2 partially colocalized with ER. In addition to serving as the virus replication site, the ER is also central to the cytopathic effects and death observed in the infected cells (Monel et al., 2017, 2019). We also found that the cells expressing NSP1, VP1a, and VP3 proteins shrank, in which the ER aggregated and distributed in perinuclear, indicating that these viral proteins may be involved in inducing cell death. In addition, the VP1b protein induced the formation of ER-derived large cytoplasmic vacuoles (Figure 2 the white arrow), which may be followed by “implosive” cell death (Monel et al., 2017). The results indicated that ALSV proteins either colocalized with ER or led to ER condensation and cell death.

### The ER colocalization of ALSV proteins in HepG2 and Vero cells

**Figure 3:**
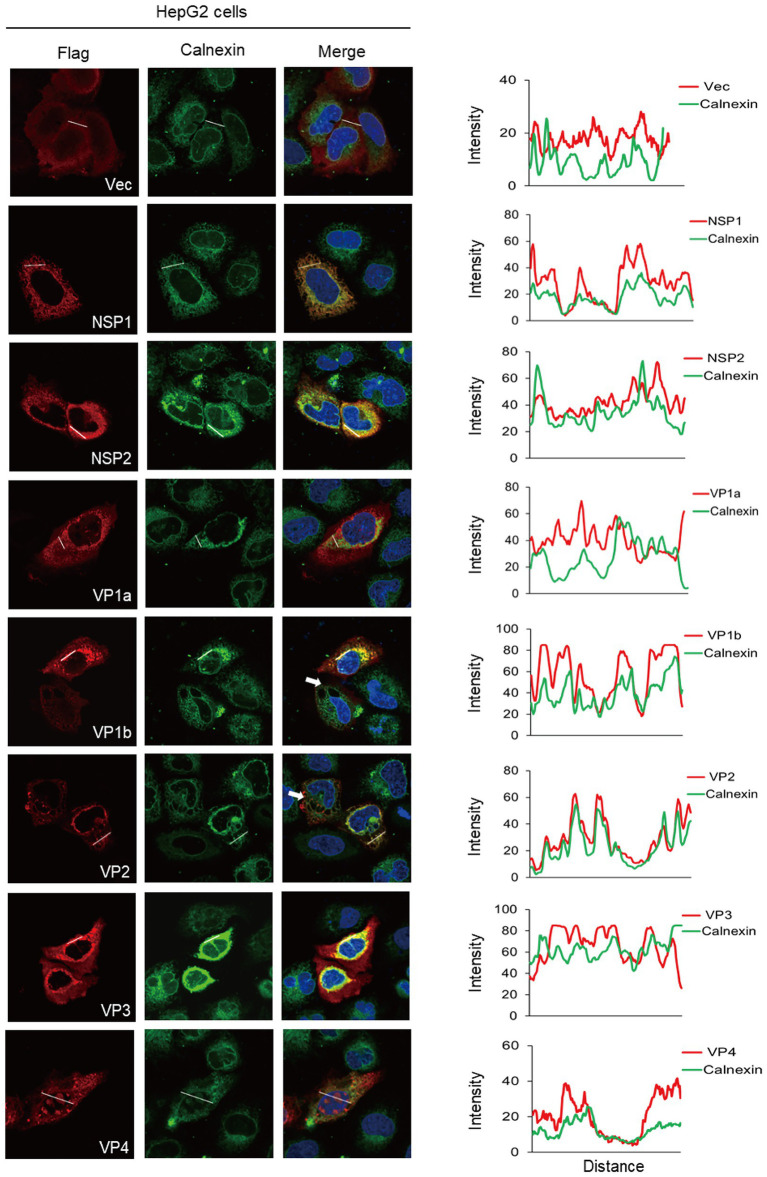
The ER colocalization of ALSV proteins in HepG2 cells. HepG2 cells were transfected with the plasmids expressing Flag-tagged viral proteins or empty vector. After 24 h, cells were immune-stained with anti-Flag antibody (red), anti-calnexin antibody (green), and DAPI (blue). The ImageJ line scan analyses are shown on the right. The distance in the x-axis represents the length of the plotted lines, and the consistency of the change trend of intensity profiles is proportional to the degree of co-localization of the two proteins.

Considering that viruses have different sensitivities to different tissue-sourcing cells, we used the same method to characterize the co-localization of ALSV proteins with ER in HepG2 and Vero cells. In HepG2 cells, the co-localization analysis demonstrated that NSP1 and VP2 were significantly co-localized with ER; NSP2, VP1b, and VP4 were partially co-localized with ER (Figure 3). Different from HEK293T cells, VP4 and calnexin were distributed in both cytoplasm and nucleus of HepG2 cells and appeared as obvious overlapping dots in the nucleus. A small amount of VP1a was also found in nucleus; none of the viral proteins caused obvious cell shrinkage (Figure 3). In addition to VP1b, VP2 also induced ER-derived large cytoplasmic vacuoles in HepG2 cells (Figure 3 the white arrow).

In Vero cells, NSP2, VP1b, VP2, and VP4 co-localized with ER, all viral proteins distributed in the cytoplasm, and NSP1 induced ER aggregated and cellular shrinking, all of which are the same as HEK293T cells (Supplementary Figure S1). Unlike HEK293T and HepG2 cells, NSP2 induced the ER-derived cytoplasmic vacuoles (Supplementary Figure S1 white arrow); VP1a, VP1b, and VP3 attenuated or disappeared ER fluorescence signal; and VP2 caused ER condensation into a punctate distribution in the cytoplasm (Supplementary Figure S1). Collectively, these results indicated that viral proteins exhibited diverse subcellular distribution and induced various ER morphological changes in different deriving cells. The NSP2, VP1b, VP2, and VP4 proteins were all co-localized with ER of these cells.

### VP1a and VP4 induce calnexin transfer into the nucleus in HepG2 cells

**Figure 4:**
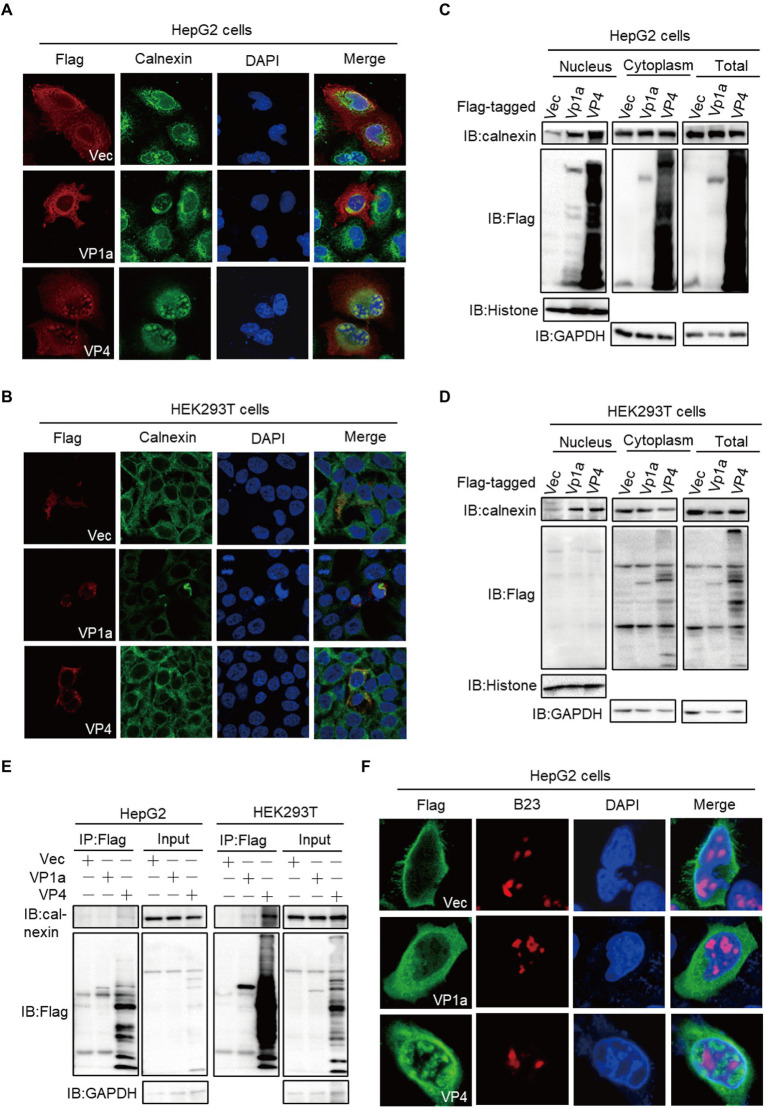
VP1a and VP4 induce calnexin transfer into the nucleus in HepG2 cells. **(A,B)** HepG2 **(A)** and HEK293T cells **(B)** were transfected with the plasmids expressing Flag-tagged VP1a, VP4 or empty vector. At 24 hpt, the cells were subjected to immunofluorescence with anti-calnexin (green), anti-Flag (red) antibodies and DAPI (blue). (C, D) HepG2 **(C)** and HEK293T **(D)** cells were transfected with the indicated plasmids. After 24 h, cells were treated using the nuclear and cytoplasmic protein extraction kit and the purified protein fractions were subjected to immunoblots with the indicated antibodies. **(E)** HepG2 cells were transfected with Flag-tagged VP1a, VP4 or vector plasmids. At 24 hpt, the cells were subjected to immunofluorescence with anti-B23 (red) and anti-Flag (green) antibodies. **(F)** HepG2 (left) and HEK293T (right) cells were transfected with Flag-tagged VP1a, VP4 or vector plasmids. At 24 hpt, anti-Flag immunoprecipitates were analyzed by immunoblots.

We found that VP1a and VP4 proteins entered the nucleus in the liver-deriving HepG2 cells but not in human embryonic kidney epithelial HEK293T cells and African green monkey kidney-deriving Vero cells. We transfected VP1a and VP4 plasmids into HepG2 or HEK293T cells for immunofluorescence staining (IFA) assay to further verify this phenomenon. The results were consistent with the previous discovery. In HepG2 cells, VP4 and calnexin entered the nucleus and accumulated as scattered circular spots. VP1a was less in the nucleus than cytoplasm while mediated calnexin transferred into the nucleus (Figure 4A). In HEK293T cells, both VP1a and VP4 were only distributed in the cytoplasm (Figure 4B). We then confirmed the localization of VP1a and VP4 in HepG2 and HEK293T cells by subcellular fractionation assays, and the results were in line with our expectations. VP1a and VP4 entered the nucleus and also induced calnexin into the nucleus in HepG2 cells (Figure 4C), whereas they did not exist in the nucleus but still mediated calnexin transferring into the nucleus in HEK293T (Figure 4D). Previously, we found that VP4 protein induced calnexin into the nucleus and co-localized with calnexin in HepG2 nucleus (Figure 4A). Therefore, we speculated VP4 might mediate calnexin nuclear-transferring through protein–protein interaction and detected whether VP4 interacted with calnexin by co-immunoprecipitation assay. Unexpectedly, neither VP1a nor VP4 bound to calnexin in HepG2 cells, whereas VP4 significantly interacted with calnexin in HEK293T cells (Figure 4E). These results indicated that VP1a and VP4 mediated calnexin nuclear-transferring independent of these interactions in HepG2 cells, and VP4 interacted with and induced calnexin into the nucleus in HEK293T cells.

The nucleus is a double-membrane-bound organelle that contains many protein-composed dot-like domains with specialized functions (subnuclear organelles), such as nucleus, Cajal bodies (CBs), and speckles [35, 36]. VP4 protein is morphologically consistent with the punctate distribution of the nucleolus, so we want to explore whether VP4 co-localize with nucleolus and plays key roles in viral protein trafficking and ribosome assembly. The HepG2 or HEK293T cells were fixed at 24 hpt and stained with anti-Flag in green and anti-B23 to visualize the nucleolus in red, and the results showed that VP1a and VP4 did not co-localize with nucleolus in both HepG2 and HEK293T cells (Figure 4F; Supplementary Figure S2), which indicates that VP1a and VP4 may associate with other domains in the nucleus, such as splicing compartments which is essential for gene splicing (Fu and Maniatis, 1992).

### NSP1 reduced mitochondria quantity by inducing mitophagy

**Figure 5:**
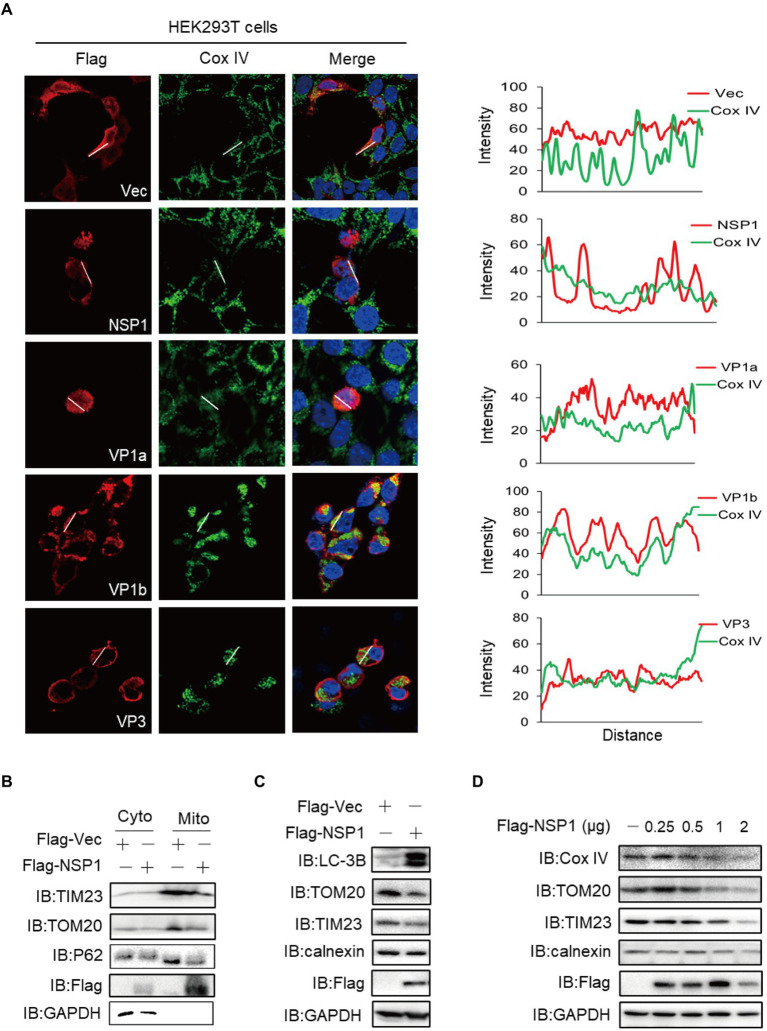
NSP1 reduced mitochondria quantity by inducing mitophagy. **(A)** HEK293T cells were transfected with the plasmids expressing Flag-NSP1, VP1a, VP1b or VP3. At 24 hpt, the cells were subjected to immunofluorescence with anti-Flag (red), anti-Cox IV (green) antibodies and DAPI (blue). The ImageJ line scan analyses are shown on the right. The distance in the x-axis represents the length of the plotted lines, and the consistency of the change trend of intensity profiles is proportional to the degree of co-localization of the two proteins. **(B)** The Flag-tagged NSP1 or vector plasmids were transfected into HEK293T cells. At 24 hpt, the cells suffered from mitochondria isolation and were measured by immunoblot with the indicated antibodies. **(C)** HEK293T cells were transfected with the indicated plasmids, 24 h later, cells proteins were analyzed by immunoblot. **(D)** HEK293T cells in a 24-well plate were transfected with the increasing amount of Flag-NSP1 (0.25, 0.5, 1and 2 μg) plasmid. At 24 hpt, the expression levels of indicated proteins were analyzed by immunoblot.

Mitochondria is an essential functional organelle, supplying energy to cells and participating in various important cellular processes, including cellular oxidative stress, calcium homeostasis, antiviral immune signaling, autophagy, and apoptosis (Vandecasteele et al., 2001; West et al., 2011; Bertero and Maack, 2018; Pickles et al., 2018). The viral infection leads to many physiological changes in host cells, including the induction of mitophagy (Zhang et al., 2018). We screened the subcellular localization of ALSV proteins to mitochondria with the marker protein Cox IV and found that with the exception of VP1b, the co-localization of other viral proteins with Cox IV was not shown (Figure 5A; Supplementary Figure S3A). Consistent with the results of ER staining, HEK293T cells shrunk when ectopic expression of NSP1, VP1a, and VP3. Additionally, we found that NSP1 could attenuate or disappear mitochondrial fluorescence signal, indicating that NSP1 specifically reduced mitochondria quantity. Mitochondria were also partially reduced or agglutinated in the cells transfected with VP1a, VP1b, and VP3 (Figure 5A). To determine whether these proteins cause mitochondrial reduction and whether they are involved in mitophagy, we first examined the effect of viral proteins on the expression of mitochondrial marker Cox IV. The results showed that NSP1, VP1a, VP1b, and VP3 proteins reduced the expression of Cox IV (Supplementary Figure S3B). Then, we transfected NSP1 into HEK293T cells and performed an immunoblot assay, and found NSP1 also inhibited the mitochondrial marker proteins TIM23 and TOM20 expression (Figures 5B,C), which was consistent with the results of the immunofluorescent assay. Simultaneously, NSP1 decreased the expression of autophagy-related protein P62 (Figure 5B) and increased the expression of microtubule-associated protein 1 light chain 3B (LC3B; Figure 5C), indicating that NSP1 can induce mitochondrial autophagy. Moreover, NSP1 dose-dependently inhibited the expression of mitochondrial marker proteins Cox IV, TOM20, and TIM23, but not the ER marker calnexin (Figure 5D). These results indicate that NSP1 reduced mitochondria quantity by inducing mitophagy.

### ALSV infection induces calnexin nuclear transfer and mitophagy

**Figure 6:**
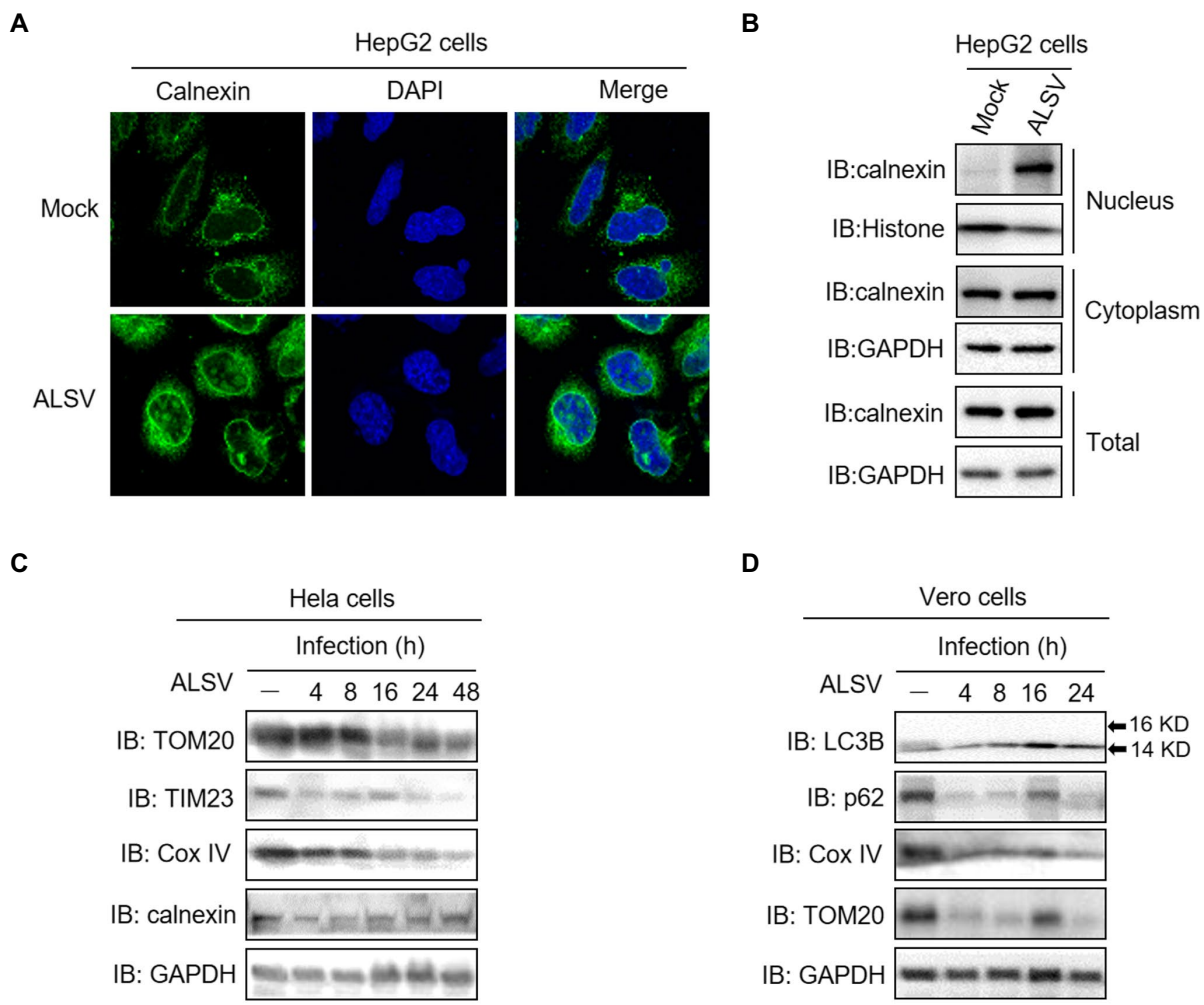
ALSV infection induces calnexin nuclear transfer and mitophagy. **(A)** HepG2 cells were infected with ALSV at MOI of 10. At 48 h post infection (hpi), the cells were subjected to immunofluorescence with anti-calnexin (green) antibody and DAPI (blue). **(B)** HepG2 cells were infected with ALSV. At 48 hpi, the cells were subjected to cell fractionation and immunoblot assay. Hela **(C)** or Vero **(D)** cells were infected with ALSV, the cells at the indicated hours post infection were analyzed by immunoblot.

We further validated our findings on a real infection model, and found that ALSV infection induced calnexin transferring into the nucleus of HepG2 cells using the immunofluorescence and cell fractionation analysis (Figures 6A,B). Additionally, ALSV infection inhibited the expression of mitochondrial marker proteins TOM20, TIM23 and Cox IV in both Hela and Vero cells, but ALSV did not affect expression of the ER marker calnexin (Figure 6C). ALSV also promoted the autophagy-related protein LC3B-II accumulation and p62 degradation (Figure 6D). These results indicated that ALSV infection induces mitochondrial autophagy.

## Discussion

ALSV usurps and manipulates the host-cellular organelles to generate a cell environment that is permissive to viral replication. Clarifying the subcellular distributions of the viral proteins will benefit us in understanding the infection and pathogenic mechanisms. In this study, we characterized the subcellular localization of ALSV proteins and found that viral proteins exhibited diverse subcellular distribution and induced various ER morphological changes in HEK293T, HepG2, and Vero cells. The nuclear transfer and co-localization of VP4 and calnexin were unique to HepG2 cells; NSP1 protein reduced mitochondria quantity by inducing mitophagy, and all the ALSV proteins did not co-localize with the Golgi apparatus (Supplementary Figure S4).

To justify why the viral proteins were located in the corresponding subcellular compartments, we predicted the subcellular localizations of viral proteins using the state-of-the-art computational methods FUEL-mLoc (Wan et al., 2017), Virus-mPLoc (Shen and Chou, 2010), and mGOASVM (Wan et al., 2012), which found that the results obtained by different methods vary widely (Supplementary Table S1). The Virus-mPLoc prediction results of all ALSV viral proteins distributed in host cytoplasm were consistent with our results, and the VP3 colocalized with host cell membrane is reasonable because it has nine transmembrane domains, which may explain the relationship between the viral protein amino acid sequences and their subcellular locations. The mGOASVM prediction results of the non-structural proteins NSP1 and NSP2 colocalized with host endoplasmic reticulum also further support our findings.

The endoplasmic reticulum, an important site for protein folding and post-translational modification, is an essential organelle for viral replication and maturation (Bagchi, 2020). After flavivirus particles enter cells, the released RNA is translated at the rough ER into a single polyprotein, which is cleaved into structural and non-structural proteins. Then, the non-structural proteins orchestrate ER membrane to form the viral replicase complex where the viral particles are produced and budded into the ER lumen (Tardif et al., 2004; Yu et al., 2006; Ambrose and Mackenzie, 2011). The functional viral proteins of ZIKV are synthesized in ER and exhibit a unique subcellular distribution in host cells, of which the E, PrM, NS2a, and NS4a proteins are co-localize with ER (Hou et al., 2017; Coyaud et al., 2018). In this study, we found that ALSV NSP1, NSP2, VP1b, VP2, and VP4 proteins were co-localized with ER in various cells which may be due to the fact that ER is the synthesis site of these viral proteins, and speculate that the non-structural proteins NSP1 and NSP2 have involved in ER membrane-associated replication complexes formation as with other flaviviruses. Virus and viral proteins are localized to ER and induce the reticular architecture remodeling, creating an ideal viral replication environment, simultaneously introducing additional burden and ER stress to host cells (Oakes and Papa, 2015). The accumulation of misfolded viral proteins in ER lumen after ZIKV infection overwhelms ER protein folding capacity, leading to ER stress and triggering the unfolded protein response (UPR) which attempts to restore the disturbed homeostasis, once the restoration failing will initiate the apoptosis or paraptosis-like cell death (Monel et al., 2017, 2019; Turpin et al., 2021). Here, we found NSP1, VP1a, and VP3 induced HEK293T cells shrinking and ER aggregating in perinuclear, suggesting that ALSV proteins may activate apoptosis response; VP1b, VP2, and NSP2 induced the form of ER-derived cytoplasmic vacuoles in various cells, indicating that ALSV proteins also activate paraptosis-like cell death.

Human hepatoma cells exhibit cell-type dependent morphology changes after flaviviruses infection, such as ZIKV induced a unique replication organelle (named zippered ER (zER) membranes) and bigger vesicles in infected hepatoma cells (Cortese et al., 2017). Here, we found that VP1a and VP4 entered the nucleus and mediated calnexin nuclear-transferring independent of their interaction, which was unique to HepG2 cells and argued for a cell type-specific role. Calnexin, a calcium ion-binding protein localized on the ER membrane is essential for flavivirus replication and assembly complexes formation. The hepatitis B virus (HBV) envelope protein requires calnexin for proper folding and transport (Prange et al., 1999), and the production of infectious dengue virions is significantly reduced when calnexin is knockdown (Limjindaporn et al., 2009). Additionally, calnexin is found in the nucleus of hepatocytes (Czubryt et al., 1996), which may explain why calnexin is distributed in the nucleus of HepG2 cells. However, the mechanisms and functions of calnexin nuclear-transferring mediated by VP1a and VP4 are not clear, and further research is needed. Although VP1a and VP4 cannot enter the nucleus in HEK293T cells, they still induce calnexin into the nucleus, and the mechanism remains to be further studied.

Mitochondria are double-membrane enclosed sub-cellular organelles that are important for viral infection (Qu et al., 2019). Flavivirus infection can remove dysfunctional mitochondria through mitophagy to promote viral replication (Ghosh et al., 2016). DENV infection induces mitochondrial elongation or fragmentation to promote viral replication (Yu et al., 2015; Chatel-Chaix et al., 2016; Barbier et al., 2017). HCV and JEV viral proteins are enriched in mitochondria and promote mitochondrial fragmentation and mitophagy (Ruggieri et al., 2014; Siu et al., 2016; Kim et al., 2017; Qu et al., 2019; Agarwal et al., 2022). In the present study, we found that NSP1 reduced the expression of mitochondrial marker proteins TOM20 and TIM23; decreased the autophagy-related protein p62, and increased the autophagy marker protein LC3B, indicating that NSP1 may induce mitophagy. The flavivirus viruses can disrupt the mitochondrial network to avoid antiviral immune activation (Chatel-Chaix et al., 2016; Du Toit, 2016; Riedl et al., 2019; Ponia et al., 2021). Therefore, NSP1 may suppress the host antiviral immune response by inducing mitophagy.

We also screened the co-localization of ALSV proteins with the Golgi apparatus with the marker protein GM130, and found that no viral proteins was co-localize with the Golgi apparatus (Supplementary Figure S4). However, it cannot be ruled out that ALSV proteins interact with the Golgi apparatus by forming the newly synthesized virus particles which are transported from ER and Golgi apparatus to the cell surface (Hansen et al., 2017).

In summary, we found ALSV proteins distributed in the cytoplasm, with the exception of VP1a and VP4 expressed in HepG2 cells. The nuclear transfer and co-localization of VP4 and calnexin independent of their interaction was unique to HepG2 cells, and the underlying mechanism still requires further study. Additionally, ALSV proteins were either co-localized with ER or induced various ER morphological changes, and NSP1 protein reduced mitochondria quantity by inducing mitophagy. Our results provide new insight into the biological functions of ALSV proteins because the subcellular distribution of viral protein hints at its possible biological function there. However, our study utilizes the surrogate expression of viral proteins, which may not reflect the real expression level upon ALSV infection and the complex environment achieved by simultaneously expressing all viral proteins.

## Data availability statement

The original contributions presented in the study are included in the article/Supplementary material; further inquiries can be directed to the corresponding authors.

## Ethics statement

The studies involving human participants were reviewed and approved by the ethics committee of the Inner Mongolia General Forestry Hospital. The patients/participants provided their written informed consent to participate in this study.

## Author contributions

QL, YZ, and ZH designed the experiments. YZ, PW, LL, BM, YH, ND, and HY conducted the experiments. Z-DW, LS, and ZH analyzed the data and conducted statistical analysis. YZ, MP, and QL drafted and revised the paper. All authors contributed to the article and approved the submitted version.

## Funding

This work was supported by grant from the National Natural Science Foundation of China (81972873), the Pearl River Talent Plan in Guangdong Province of China (2019CX01N111), and the Scientific and Technological Research Projects of Guangzhou (202103000008).

## Conflict of interest

The authors declare that the research was conducted in the absence of any commercial or financial relationships that could be construed as a potential conflict of interest.

## Publisher’s note

All claims expressed in this article are solely those of the authors and do not necessarily represent those of their affiliated organizations, or those of the publisher, the editors and the reviewers. Any product that may be evaluated in this article, or claim that may be made by its manufacturer, is not guaranteed or endorsed by the publisher.
