# Supplementary material for: Characterization and subcellular localization of Alongshan virus proteins
Source: Front Microbiol. 2022 Sep 27;13:1000322. doi: 10.3389/fmicb.2022.1000322 (PMC9551281; doi:10.3389/fmicb.2022.1000322)
Supplement: Supplementary file 2 [file Data_Sheet_1.PDF]

Supplementary material for

Characterization and subcellular localization of Alongshan virus proteins

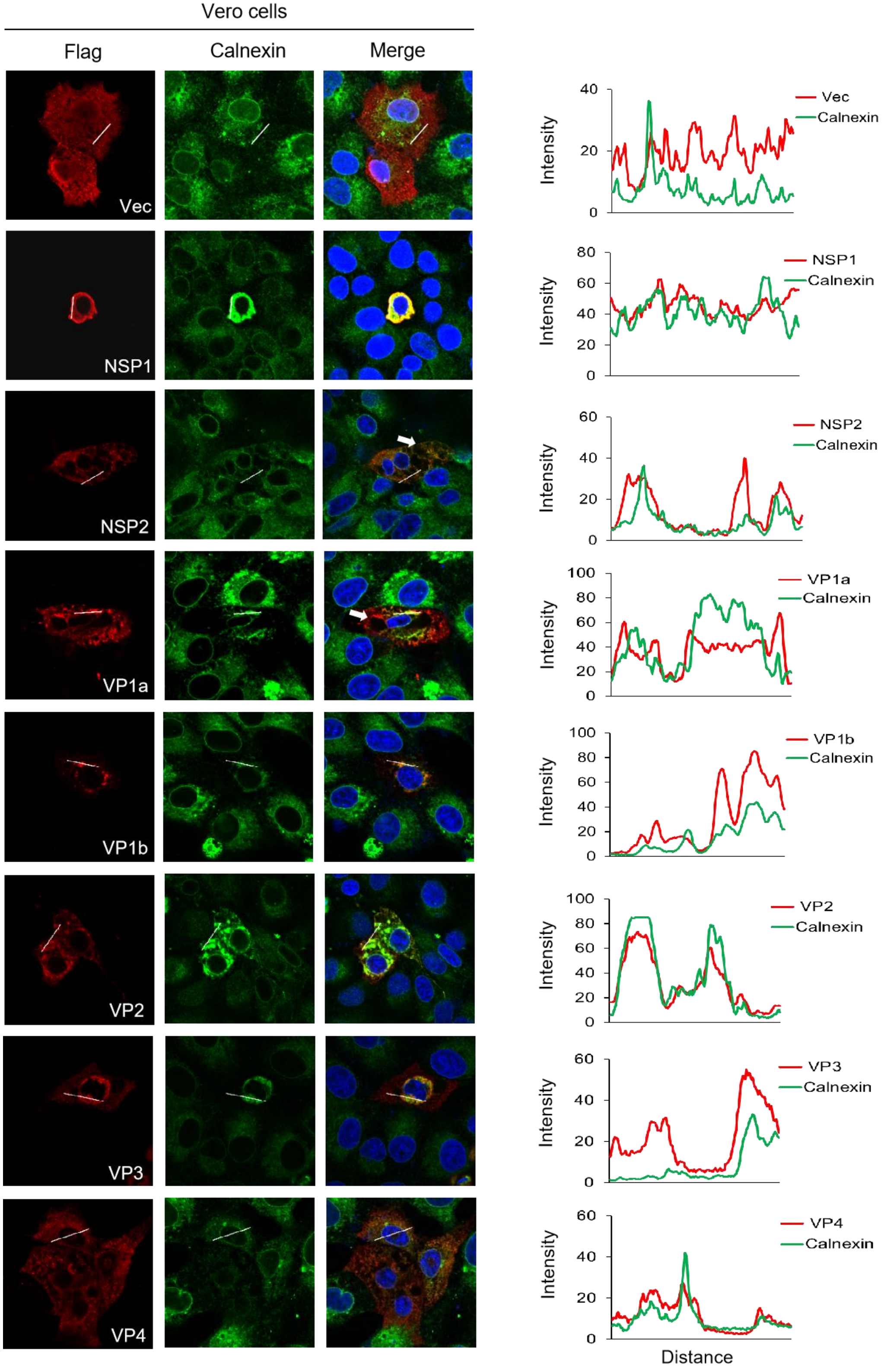

Supplementary Figure 1. The ER subcellular localization of ALSV proteins in Vero cells. Vero cells were transfected with the plasmids expressing 0.25 μg Flag-tagged ALSV proteins or vector in 24-well plate. After 24 h, the cells were immune-stained with anti-Flag (red), anti-calnexin (green) antibodies and DAPI (blue). (Right) Image J line scan analysis of the intensity profiles of the viral protein (red) and the calnexin (green) along the plotted lines.

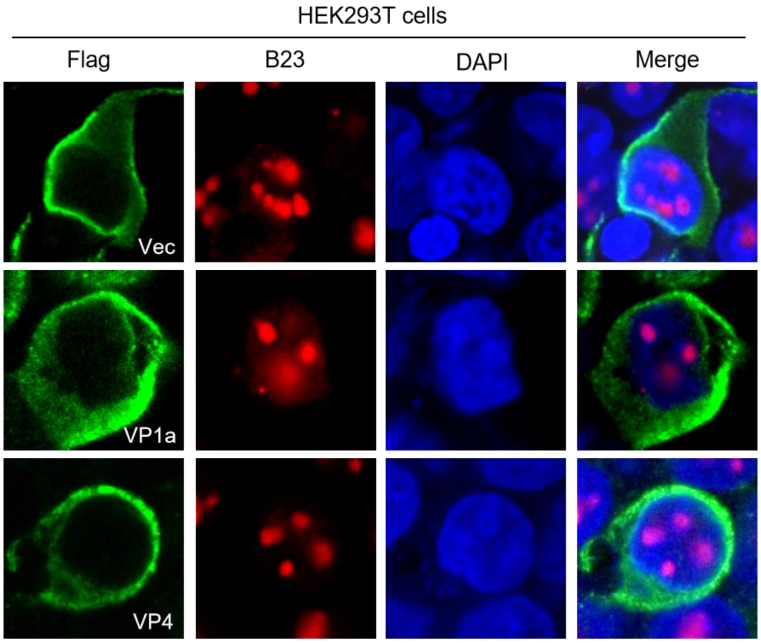

Supplementary Figure 2. VP1a and VP4 did not co-localize with nucleolus in HEK293T cells. HEK293T cells were transfected with the plasmids expressing 0.25 μg Flag-VP1a, VP4 or vector in 24-well plate. At 24 hpt, the cells were subjected to immunofluorescence with anti-B23 (red), anti-Flag antibodies (green) and DAPI (blue).

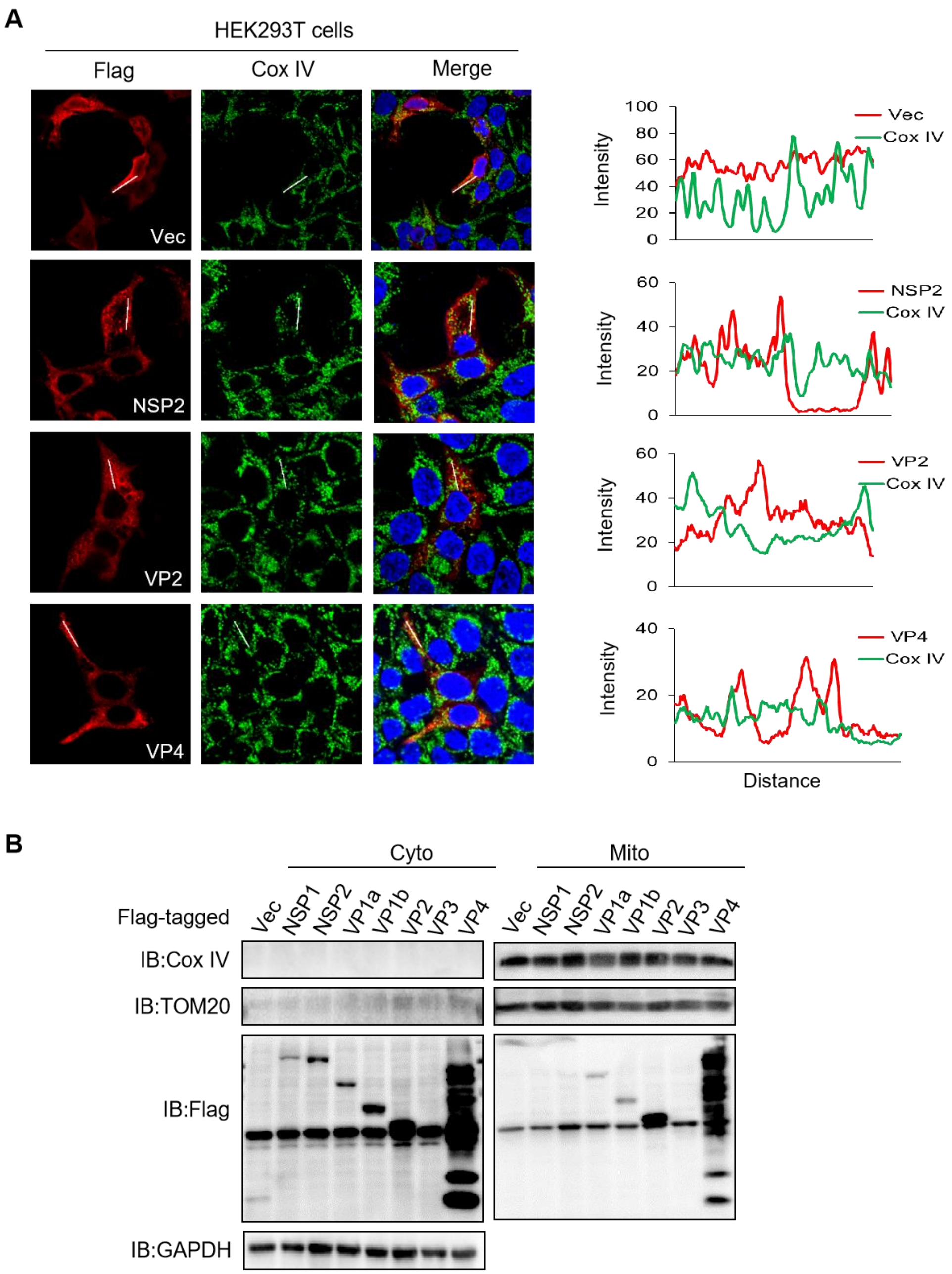

Supplementary Figure 3. The mitochondria subcellular localization of ALSV proteins in HEK293T cells. (A) HEK293T cells were transfected with Flag-NSP2, VP2, VP4 or vector plasmids in 24-well plate. At 24 hpt, the cells were subjected to immunofluorescence with anti-Cox IV (green), anti-Flag (red) and DAPI solution (blue). The Image J line scan analysis are shown on the right. **(B)** HEK293T cells were transfected with the plasmids expressing 0.25 μg Flag-tagged ALSV proteins in 24-well plate. The cells were treated with Cell Mitochondria Extraction Kit according to the manufacturer's instructions and were measured by immunoblot with the indicated antibodies. GAPDH was used as a loading control.

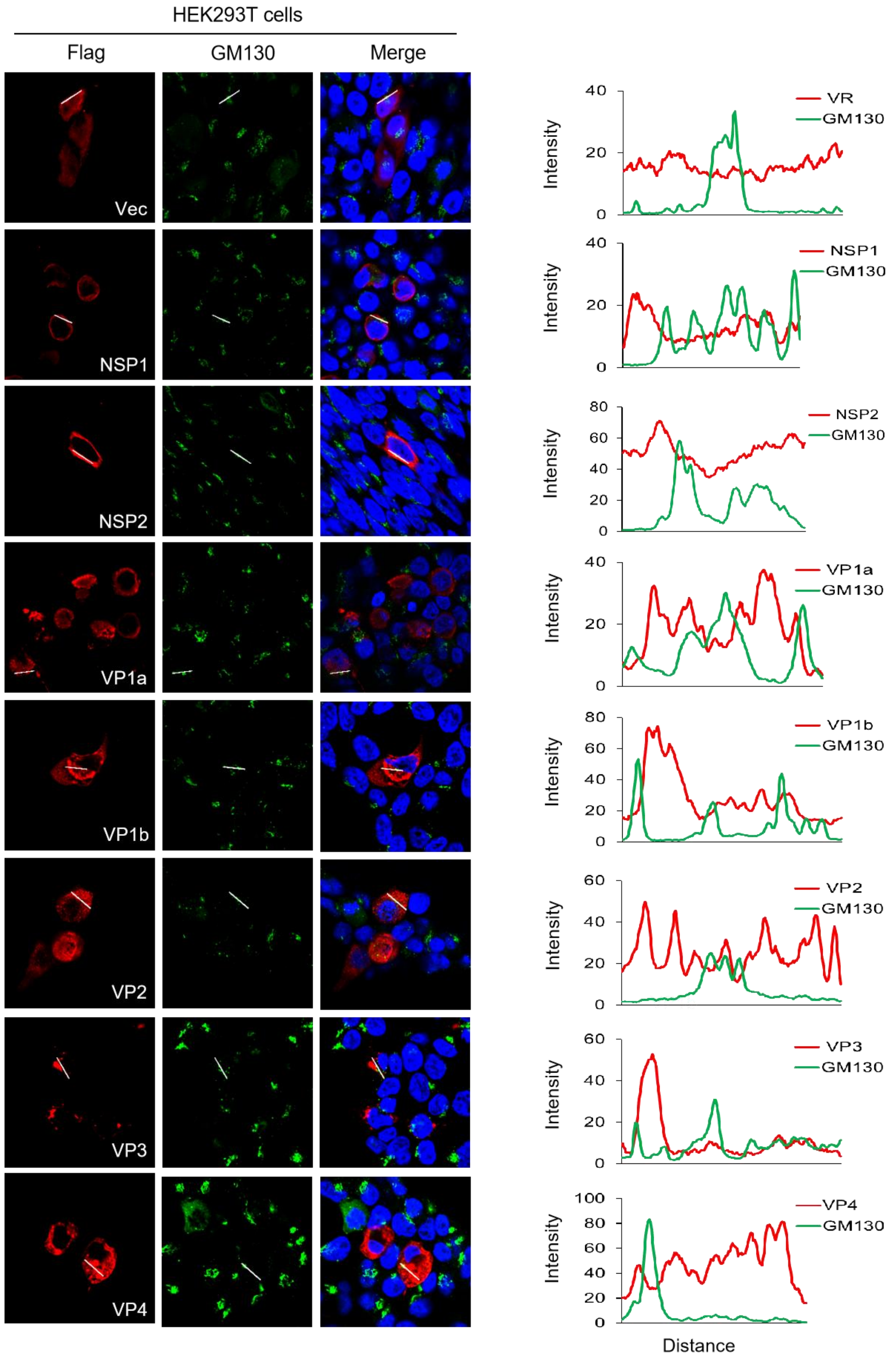

Supplementary Figure 4. The Golgi apparatus subcellular localization of ALSV proteins in HEK293T cells. HEK293T cells were transfected with Flag-tagged ALSV proteins or vector plasmids in 24-well plate. At 24 hpt, the cells were subjected to immunofluorescence with anti-GM130 (green), anti-Flag (red) and DAPI solution (blue). The Image J line scan analysis are shown on the right.
